# Supplementary material for: Association between Dental Scores and Saliva Uremic Toxins
Source: Toxins (Basel). 2023 Nov 20;15(11):666. doi: 10.3390/toxins15110666 (PMC10674306; doi:10.3390/toxins15110666)
Supplement: Supplementary file 1 [file toxins-15-00666-s001.zip › toxins-2587115-supplementary.pdf]

Association between dental scores and saliva uremic toxins

Claire Rigothier, Sylvain Catros, Antoine Bénard, Johan Samot, Olivier Quintin, Christian Combe, Islam Larabi, Ziad Massy and Jean-Claude Alvarez

Table S1. p-values of univariate analyses of the association between dental scores and levels of uremic toxins detected in saliva of CKD patients (Nephrodent Study).

Saliva uremic toxins	DMFT score	FST score	OHIS score	CPITN score
TMAO	0.23	0.09	0.74	0.04*
Tyrosine	0.10	0.86	0.54	0.75
Phenylalanine	0.09	0.8	0.97	0.58
Tryptophane	0.22	0.59	0.55	0.28
Kynurenine	0.35	0.37	0.84	0.32
Hippuric acid	0.005*	0.02*	0.99	0.29
Phenylacetylglutamine	0.046*	0.11	0.07	0.39
Indoxyl sulfate	0.20	0.17	0.47	0.11
p-cresyl-sulfate	0.11	0.35	0.18	0.07
Indol-3-acetic acid	0.38	0.1	0.87	0.21
CMPF	0.10	0.54	0.24	0.94

Table S2. p-values of multivariate analyses of the association between dental scores and levels of uremic toxins detected in saliva of CKD patients (Nephrodent Study).

Saliva uremic toxins	DMFT score	FST score	OHIS score	CPITN score
**TMAO**		0.05*		0.01*
Sexe		0.18		0.86
Age		0.11		0.055*
BMI		0.11		0.98
Alcohol/Tabacco		0.43/0.27		0.18/0.01*
CKD stage		0.25		0.32
**Tyrosine**	0.11			
Sexe	0.65			
Age	0.0006*			
BMI	0.03*			
Alcohol/Tabacco	0.61/0.9			
CKD stage	0.53			
**Phenylalanine**	0.11			
Sexe	0.69			
Age	0.0008*			
BMI	0.02*			
Alcohol/Tabacco	0.5/0.88			
CKD stage	0.56			
**Hippuric acid**	0.07	0.018*		
Sexe	0.57	0.33		
Age	0.002*	0.14		
BMI	0.05*	0.39		
Alcohol/Tabacco	0.82/0.74	0.32/0.29		
CKD stage	0.71	0.12		
**Phenylacetylglutamine**	0.12	0.06	0.03*	
Sexe	0.62	0.3	0.05*	
Age	0.003*	0.09	0.72	
BMI	0.01*	0.1	0.17	
Alcohol/Tabacco	0.34/0.92	0.41/0.24	0.76/0.21	
CKD stage	0.76	0.15	0.58	
**Indoxyl sulfate**		0.14		0.04*
Sexe		0.3		0.78
Age		0.14		0.06
BMI		0.11		0.96
Alcohol/Tabacco		0.38/0.3		0.13/0.03*
CKD stage		0.26		0.42
**p-cresyl-sulfate**	0.08		0.11	0.17
Sexe	0.75		0.06	0.76
Age	0.001*		0.8	0.13
BMI	0.01*		0.14	0.81
Alcohol/Tabacco	0.5/0.89		0.77/0.42	0.2/0.06
CKD stage	0.76		0.72	0.3
**CMPF**	0.07			
Sexe	0.73			
Age	0.001*			
BMI	0.01*			
Alcohol/Tabacco	0.5/0.89			
CKD stage	0.76			
